# Synthesis, characterizations and disinfection potency of gelatin based Gum Arabic antagonistic films

**DOI:** 10.1038/s41598-025-90431-3

**Published:** 2025-03-10

**Authors:** Marwa M. Eltarahony, Mohamed A. Elblbesy, Taha A. Hanafy, Bothaina A. Kandil

**Affiliations:** 1 Environmental Biotechnology Department, Genetic Engineering and Biotechnology Research Institute, City of Scientific Research and Technological Applications (SRTA-City), New Borg El-Arab City, 21934 Alexandria Egypt; 2 Department of Medical Laboratory Technology, Faculty of Applied Medical Sciences, University of Tabuk, Tabuk, Saudi Arabia; 3 Department of Medical Biophysics, Medical Research Institute, Alexandria University, Alexandria, 21561 Egypt; 4 Nanotechnology Research Laboratory, Department of Physics, Faculty of Science, University of Tabuk, Tabuk, Saudi Arabia; 5 Physics Department, Faculty of Science, El Fayoum University, Fayoum, Egypt; 6 Department of Radiological Science and Medical Imaging, Faculty of Allied Medical Science, Pharos University, Alexandria, Egypt

**Keywords:** Polysaccharide/polymer, Protein, Multidrug-resistant microorganisms, Coliforms, Wastewater treatment, Antimicrobial, Municipal effluents, Industrial effluents, Biotechnology, Microbiology, Environmental sciences, Environmental social sciences, Health care, Materials science

## Abstract

Water-borne infections are considered as one of the major risky concerns regarding the sanitary state of water bodies dedicated to drinking water supply. Therefore, the employment of environmentally benign materials in water/wastewater treatment is an indispensable aspect to solve the water crisis problem in an eco-friendly and economic manner. This study describes the synthesis, characterization, and disinfection potency of different formulas of gelatin-based Gum Arabic composites, for the first time. SEM, XRD, FTIR, *ζ-*potential, and swelling tests were used to assess their physicochemical properties, which revealed the enhanced compatibility and miscibility with increasing Gum Arabic concentration. The formula of GEL/50%GA showed more homogenously distributed pores as visualized by SEM with noticeable shifts in the characteristic FTIR-band and more negatively charged surface, reflecting the considerable stability as indicated by ζ-potential. Besides, it also had superior hydrophilic and swellability levels. Interestingly, the results of antimicrobial activity showed the susceptibility of broad-spectrum microbes against examined composites, especially with elevating the concentration of Gum Arabic incorporated in the composite. As a natural alternative disinfectant, the as-prepared composites (3 and 10% W/V) were evaluated in the disinfection of real wastewater samples. The results revealed that GEL/50%GA (10% W/V) exhibited a noticeable reduction in total plate count by 45.62 ± 1.48% and 37.48 ± 1.63% and in coliforms by 58.43 ± 2.07% and 40.88 ± 2.24% for municipal and industrial effluents, respectively. However, the microbial metabolic activity via MTT assay was diminished by more than 50% in both effluents; denoting the efficient inhibiting capability of GEL supplemented with GA films in restricting microbial viability even in unculturable microbes. Overall, the antagonistic activity of examined composites offers promising insights for recruitment in different disciplines such as anti-biofouling membranes, food coating, dietary supplements, wound healing, and drug delivery.

## Introduction

Water is the blue artery of life; it touches inextricably the three pillars of sustainable development (i.e., social, environmental, and economic)^1^. So, it fosters healthy ecosystems and manages economic growth which subsequently supports political stability. However, climate change, either natural or human-induced, represents a great threat influencing profoundly the physical aspects of water security and water abundance. Let al.one, quick industrialization and continuous urbanization, which polluted drinking water resources and destroyed their quality. Therefore, clean water, sanitation, and hygiene are the most prerequisite basic stones for human health and a safe eco system^2^.

Generally, the conventional water purification process comprises a combination of physical, chemical, and biological stages that are devoted to eliminating water contaminants of floating solids, organic/inorganic matter, and microbial pathogens. Therefore, various methods were recognized and frequently utilized to implement this target such as sedimentation, coagulation/flocculation, adsorption, filtration, disinfection, reverse osmosis, ion exchange, electrodialysis, etc^3^. Substantially, water disinfection symbolizes the vital barrier against pathogen infections and epidemic dissemination. Thus, the efficiency of this stage is considered as a crucial redline in guaranteeing the safety of supplied water to public health. Popularly, chlorination, ozonation, photocatalysis, ultraviolet irradiation, ultrasonication, microwave and electrochemical disinfection are the most commonly applied means to disinfect water systems. Despite their effectiveness, the generation of disinfection by-products and resistant microbes, unfavorable water taste/odor, high-energy consumption, special instrumental handling, long-time and high-cost techniques are considered the major obstacles in their wide application^4^.

Hence, composites have piqued the interest of technologists and industrialists owing to their functionalization, enhanced surface area, chemical accessibility, etc. Arguably, such a combination of two or more different kinds of materials with different properties would in turn produce a new product with versatile properties dislike the parent individual materials but a new material with improved properties^3^. In this regard, engineered materials (e.g., metals, metal oxides, polymers, carbon-based materials, fibers, etc.) were the materials of choice, however, their biocompatibility and cytotoxicity implications are the fundamental concerns^5,6^. Subsequently, all insights directed toward utilizing natural materials (e.g., gelatin, Gum Arabic, cellulose, chitosan, etc.), which could compensate for the limitations of the others^4,7,8^.

Gelatin (GEL) is a natural biodegradable polymer derived from collagen. Due to its excellent emulsification, high stabilizing activity, and high crosslinking activity has various environmental applications^9,10^. Nonetheless, three-dimensional cell culture, tissue engineering scaffolds and fat replacement in the food industry are grouped among other applications^11–13^. There are two types of gelatin A, and B. Type A is an acidic treatment gelatin. Type B is an alkaline treatment gelatin found in bovine hides^14^. It has amphoteric behavior due to its functional amino acid group, terminal amino acids, and carboxyl group. It is made of glycine, proline, hydroxyproline, glutamic acid, alanine, arginine, and aspartic acid, its chemical structure contains different polypeptide chains such as α, γ, and β^15,16^.

On the other hand, Gum Arabic (GA) is a polysaccharide containing galactose, rhamnose, glucuronic acid and arabinose residues^17,18^. GA has a low viscosity effect and is highly soluble in water. It is received enormous interest in synthesizing the most active green nontoxic and biocompatible crosslinking agents, which have been used in the formation of hydrogel^19,20^. GA has a high molecular weight which prevents it from evaporation, and reduces environmental toxicity. GA has a positive effect on some physiological functions such as renal function and intestinal absorption^21–23^.

Protein /polysaccharide interaction is increasingly recognized as playing a key role in multiple biotechnology applications. These applications include the development of bioactive delivery systems, the formation of biopolymers, and food emulsion stabilizers^24,25^. The interactions between two mixed biopolymers may be attractive or repulsive, depending mainly on total biopolymer concentration, ionic strength, and the protein-to-polysaccharide ratio^26,27^. Attractive interactions may lead to the formation of a two-phase system, which is called complex coacervation or precipitation for the separation of liquid- or solid-state insoluble complexes^28^. As antibacterial agents or texture enhancers, gelatin, and Gum Arabic were investigated in previous studies, which focused on studying their effect on autonomous behavior (i.e., not in composite state), in some industrial applications^19,29,30^. Hence, in the current study, we set out the possibility of recruiting gelatin films supported by Gum Arabic in the treatment of wastewater. The negatively charged GA with different concentrations was complexed with positively charged GEL in different ratios; followed by determining their physicochemical properties by various characterization techniques. Ultimately, their antimicrobial activity and disinfection potency were evaluated, which was not formerly detected in previous studies, to our knowledge.

## Materials

The gelatin type A (GEL) and Arabic gum (GA) that were used in this study were purchased from Kose Chemical Co-Ltd, Japan. Mueller-Hinton agar and Sabouraud Dextrose Agar media were obtained from HiMedia (India). While, 3-[4, 5- dimethylthiazol-2-yl]-2, 5-diphenyltetrazolium bromide (MTT), and Dimethyl sulfoxide (DMSO) were purchased from Sigma-Aldrich, Germany. Besides, Hydrochloric acid (HCL) and sodium hydroxide (NaOH) were obtained from Merck-Germany.

## Methods

### Preparation

Gum Arabic (GA) was sundried and grained using a serrated disk grinder to obtain small-sized particles. An aqueous solution of gelatin was prepared by dissolving one gram of GEL in 100 ml of triply distilled water at 80°C. GA solution was obtained by adding the proper weight of GA_x_ (where x = 5, 10, 15, 20, 25, and 50 wt%) in 50 ml of triply distilled water at 60°C and was added to the gelatin solution to form (gelatin/GA_x_) composites. The mixture solutions were stirred at 60°C for 4 h until homogeneous mixing was completed. The composition of GA in the mixture solution of gelatin/GA_x_ was x = 0, 5, 10, 15, 20, 25, and 50 wt%. The mass fraction of GA, (wt%) was calculated according to the following equation:

1 $$\:\omega\:\left(wt.\:\%\right)=\:\frac{{\omega\:}_{GA}}{{\omega\:}_{GEL}+{\omega\:}_{GA}}\times\:100$$

Where $\:{\omega\:}_{GA}$and $\:{\omega\:}_{GEL}\:$represent the weights of GA and GEL, respectively. The final solution of the GEL/GA_x_ blend was poured into polypropylene dishes and dried at 40^o^C on a leveled plate for 3 days until the solvent was completely evaporated. The flexible uniform and transparent films, with a thickness of 0.1 mm, had been obtained and kept in the desiccators for further characterization.

### Characterization of GEL/GA

Scanning electron microscope SEM (ZEISS, Germany) was used in the morphological study of the prepared samples with an accelerated voltage of 10 kV. The structure of the obtained samples was analyzed by the XPERT-PRO diffractometer system. The chemical composition of the prepared samples was tested by Fourier transform infrared (FTIR) spectroscopy. FTIR spectrum of the prepared samples was recorded by FTIR (Bruker, Germany) in the region of 4,000 to 500 cm^− 1^. Zeta potential was measured via dynamic light scattering type (ZetasizerNano -ZS90, Malvern instruments, U.K.) apparatus equipped for protein size measurement sensitivity. For X-ray diffraction (XRD), measurements were performed at a scattering angle of 90° from two different directions, at room temperature (25 ± 1 °C). Measurements for each sample were taken in triplicates.

### Water affinities

The percentage of the total soluble matter (TSM) of the film was expressed as the percentage of dry film solubilized after immersion in water. The film specimens (5 cm^2^) were weighed and shaken with distilled water (25 mL, 30 ± 1 ºC, and 170 rpm). Undissolved residues were removed every 30 min from the water and dried (105 ºC, 24 h). The initial dry matter content of the films was determined by drying at 105ºC for 24 h and used to calculate TSM. Pre-weighed films (5 cm^2^) were immersed in distilled water (25 mL, 30 ± 1 ◦C, 1 min) and used in determining the swelling (SW). Swollen films were weighed, after blotting the surface gently with filter paper until equilibrium was reached. SW was calculated as a percentage of water absorbed by the sample. The tests were performed in triplicate.

### Antimicrobial activity of as-prepared films formulated of GEL and its combination with GA

#### Disc diffusion method

The effectiveness of gelatin (GEL) and its Gum Arabic (GA) combination formulas were examined as antimicrobial agents against some human pathogenic microbes (prokaryotic and eukaryotic) using disc diffusion assay. Initially, 6 mm discs (0.1 gm) of GEL film with different GA content (5, 10, 15, 20, 25, and 50%) were sterilized using a UV lamp for 30 min. Mueller-Hinton agar medium with the following ingredients (g/L): beef extract, 2; casein hydrolysate, 18; starch, 2; agar, 18; pH, 7.2 ± 0.2 and Sabouraud Dextrose Agar (4% Dextrose, 1% peptone, and 1.8% agar, pH = 5.6 ± 0.2) were utilized for bacterial and fungal growth, respectively. Both media were prepared according to the manual instructions, sterilized at 15 psi and 121 °C for 20 min by autoclaving, and poured into sterile Petri dishes till solidification. Thereafter, about 100 µL (1.5 × 10^6^ CFU/mL) fresh suspensions of (*Pseudomonas aeruginosa* (ATCC 15442) and *Klebsiella pneumonia* (ATTC 700603) as paradigms of Gram-negative bacteria; *Bacillus cereus* (ATCC 33019) and *Staphylococcus aureus* (ATCC 29213), as Gram-positive bacteria, while *Candida albicans* (ATCC 10231) and *Aspergillus bracelleuse* (ATCC 16404)), as examples on unicellular and filamentous fungi, respectively, were incubated at 37 °C for 24 h. Whereas, the fungal plates were incubated at 25 °C for 48 h. After incubation, the antagonistic activity of examined formulas was determined by measuring inhibition zones (mm). Each test was conducted in triplicate and the data were expressed as replicates mean ± standard error of the mean (SEM).

#### Disinfection of real wastewater samples

The potentiality of the GEL and its GA combination formulas in diminishing the microbial load was studied in two real effluent samples. One of them was municipal and the other one was an industrial wastewater sample; their physicochemical properties were determined previously according to^31^. Two sterilized doses (3 and 10% W/V) of the as-prepared GEL and its modified formulas were mixed well with 100 ml of Plate Count Agar (PCA) and violet Red Bile Agar (VRBA) and poured into sterile Petri plates, to determine the total plate count (TPC) and coliform count, respectively. After plate solidification, approximately 1 mL of serially diluted effluents were spread over polymers-containing plates and polymers-free plates (as controls), which thereafter, incubated at 30 °C for 48 h. The formed separate colonies were counted^32^. After incubation, the colony count was determined, and the results were expressed as CFU/mL. The disinfection potency was calculated according to the following equation^33^.

2 $$\:\mathbf{D}\mathbf{i}\mathbf{s}\mathbf{i}\mathbf{n}\mathbf{f}\mathbf{e}\mathbf{c}\mathbf{t}\mathbf{i}\mathbf{o}\mathbf{n}\:\mathbf{p}\mathbf{o}\mathbf{t}\mathbf{e}\mathbf{n}\mathbf{c}\mathbf{y}\:\left(\mathbf{\%}\right)=\frac{\text{N}\text{u}\text{m}\text{b}\text{e}\text{r}\:\text{o}\text{f}\:\text{c}\text{o}\text{l}\text{o}\text{n}\text{i}\text{e}\text{s}\:\text{i}\text{n}\:\text{u}\text{n}\text{t}\text{r}\text{e}\text{a}\text{t}\text{e}\text{d}\:\text{s}\text{a}\text{m}\text{p}\text{l}\text{e}\text{s}-\text{N}\text{u}\text{m}\text{b}\text{e}\text{r}\:\text{o}\text{f}\:\text{c}\text{o}\text{l}\text{o}\text{n}\text{i}\text{e}\text{s}\:\text{i}\text{n}\:\text{t}\text{h}\text{e}\:\text{t}\text{r}\text{e}\text{a}\text{t}\text{e}\text{d}\:\text{s}\text{a}\text{m}\text{p}\text{l}\text{e}}{\text{N}\text{u}\text{m}\text{b}\text{e}\text{r}\:\text{o}\text{f}\:\text{c}\text{o}\text{l}\text{o}\text{n}\text{i}\text{e}\text{s}\:\text{i}\text{n}\:\text{t}\text{h}\text{e}\:\text{u}\text{n}\text{t}\text{r}\text{e}\text{a}\text{t}\text{e}\text{d}\:\text{s}\text{a}\text{m}\text{p}\text{l}\text{e}}\times\:100$$

To the same extent, the metabolic activity of microbial load in both effluents before and after the treatment of GEL and its GA combination formulas was determined using the MTT method. Briefly, 100 µL (0.25 mg/mL) of 3-[4, 5- dimethylthiazol-2-yl]-2, 5-diphenyltetrazolium bromide (MTT) was added to overnight incubated untreated and (3 and 10% W/V) polymers-treated effluents, followed by gentle shaking and incubation in darkness at 37 °C for 3 h. Subsequently, the solution was decanted and 200 µL of DMSO (2%) was mixed to dissolve insoluble purple formazan, which is generated owing to the metabolic activity of living cells and their enzymatic reduction of tetrazolium salt. The absorbance of treated and control (untreated) samples was measured at 570 nm. The inhibition percentage was calculated from the following equation:

3 $$\:\text{I}\text{n}\text{h}\text{i}\text{b}\text{i}\text{t}\text{i}\text{o}\text{n}\text{\%}=\frac{{A}_{1}}{{A}_{0}}\times\:100$$

Where A_0_ is the absorbance of control (untreated) samples and A_1_ is the absorbance of treated samples.

### Statistical analysis

The results were expressed as mean ± SEM; Tukey posthoc analysis of variance (ANOVA) was used to determine the significant difference between different treatments either a significant difference (*p* < 0.05) or non-significant (*p* > 0.05), via Graphpad Instat software.

## Results and discussion

### Preparation and characterization of GEL/GA films

**Fig. 1:**
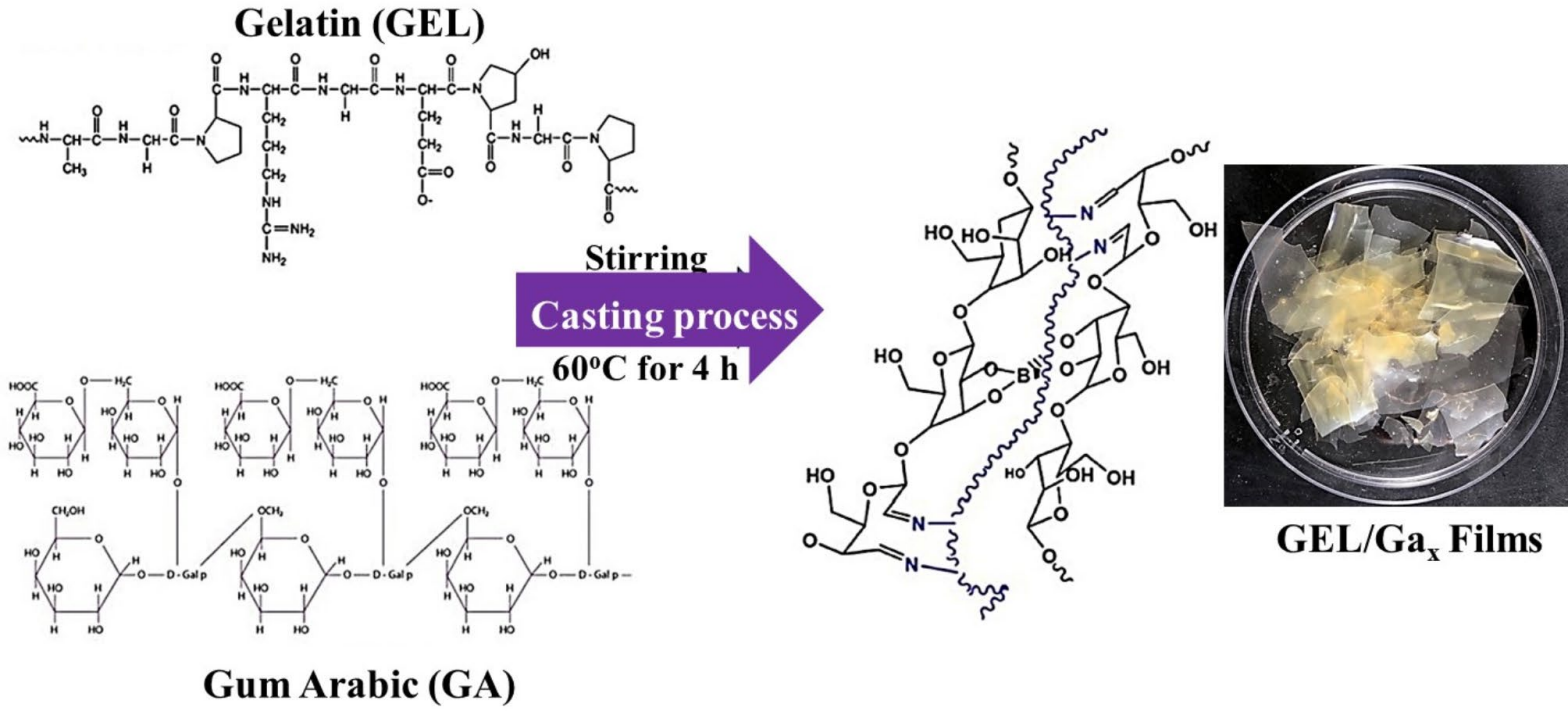
Schematic representation of GEL/GA_x_ composites.

**Fig. 2:**
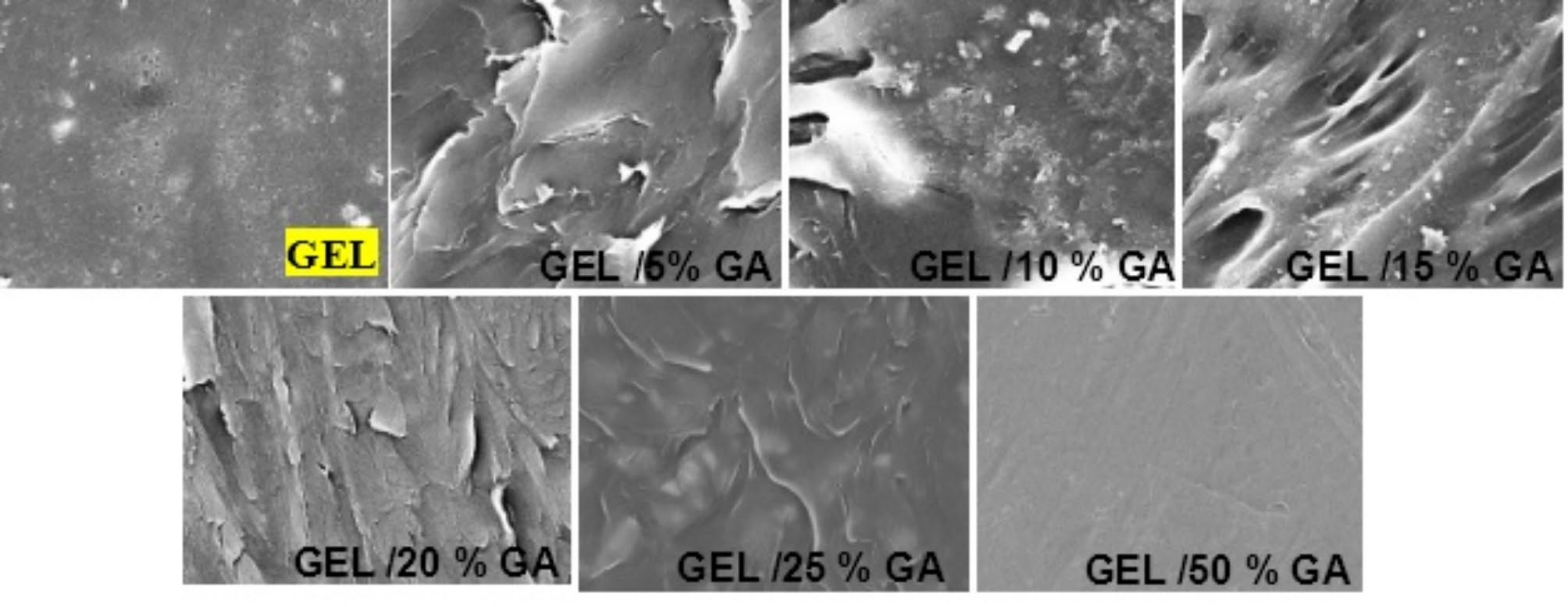
SEM micrographs of the prepared GEL/GA_x_ films observed at 5000X and 20.0KV.

Gelatin was modified to enhance the number of amino groups that contained, as shown schematically in Fig. 1 for GEL/GAx composites. The characteristics of GEL and GA were quite distinct. GA lacked the gelling properties of gelatin. Gelling in gelatin is caused by strong hydrogen bonding between the carboxyl and amino groups. Scanning electron microscope (SEM) characterization was employed to examine the morphology, distribution, and particle shape of gelatin/GA_x_ (x = 0, 5, 10, 15, 20, 25, and 50 wt%). It provides valuable information about the size and shape of the material used^1^. SEM micrographs of GEL/GA_x_ composites are shown in Fig. 2a-g. SEM of GEL/GA_x_ composites reveals good incorporation of GA inside the gelatin structure with dispersions. The whitening of the prepared gelatin/GA_x_ copolymers was increased by increasing the GA ratio within the gelatin structure. Also, the surface of the membranes was dense, and no obvious phase separation was observed for all gelatin/GA_x_ samples. Consequently, good compatibility and miscibility between gelatin and Arabic gum molecules were obtained^2,3^. The crosslinking formation between OH groups of GA and the free amino groups (NH_2_) of gelatin can be expected. Moreover, the hydrogen bonding between the functional groups of both gelatin and GA molecules plays an important role in the crosslinking process^4^. A clear effect on the morphology of GEL surface was observed with the increase of GA ratio within the polymeric sample from 5, 10, 15, 20, 25, and 50 wt%, respectively. From Fig. 2, GEL/GA_x_ (x = 5 wt%) showed the smallest pores on the surface. However, with the increase of gelatin ratio from 10 to 50 wt% within the polymeric material, the pore diameter was found to improve, and pores were more homogenously distributed.

**Fig. 3:**
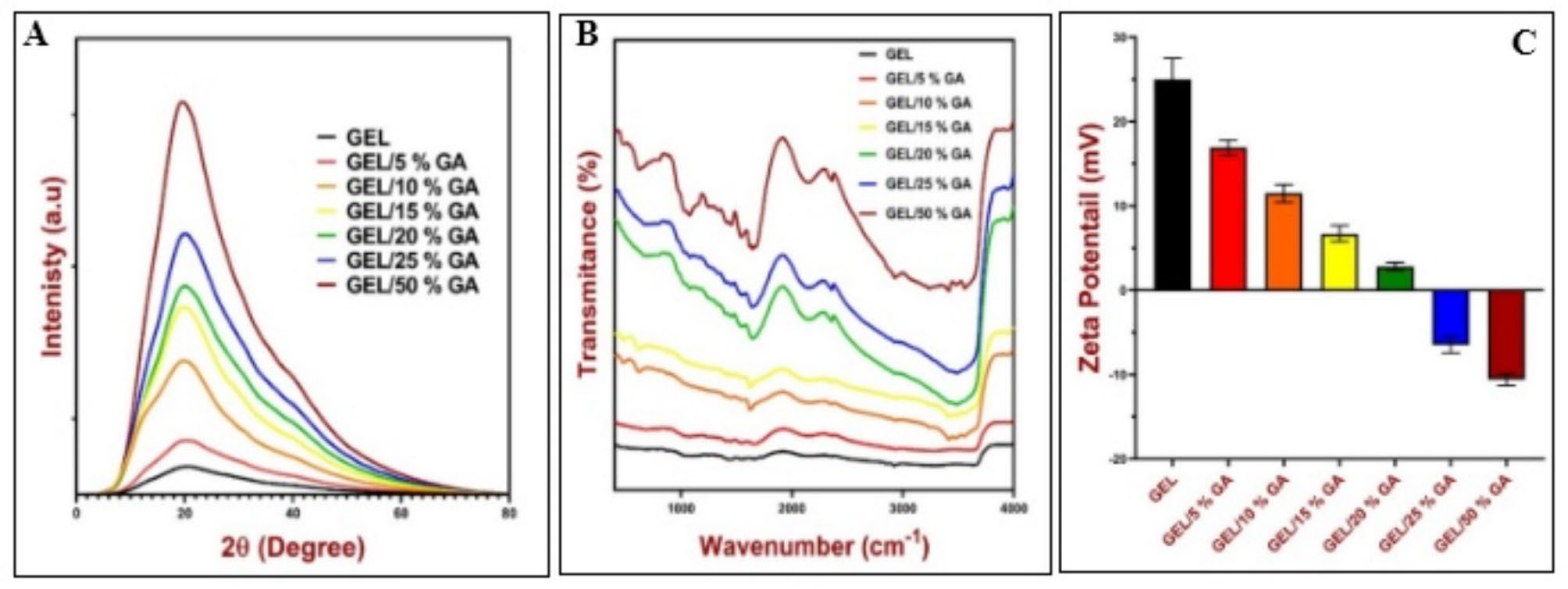
Characterstic features of the prepared GEL/GA films. (**A**) - XRD, (**B**) - FTIR and (**C**) - Zeta potential.

Besides, GEL/GA_x_ composites that contain different GA ratios were subjected to XRD for better understanding of their chemical structures. XRD patterns of (GEL/GA_x_), (x = 0, 5, 10, 15, 20, 25, and 50 wt%) were shown in Fig. 3-A. The figure shows a broad peak at 20° for pure gelatin. This peak is attributed to the triple helix structure of gelatin, and it reveals the existence of the amorphous nature of gelatin^1^. XRD patterns exhibited the same behavior for all GEL/GA_x_ samples. However, the broadness and intensity of the diffraction peak of GEL/ GA_x_ samples increased while increasing the concentration of GA within the copolymer samples. These results reveal that gelatin has a semi-crystalline structure with both crystalline and amorphous structures^34,35^. The crystalline nature of gelatin is assigned to the presence of strong inter and intra-molecular hydrogen bonding between amino (NH_2_) and hydroxyl groups (OH) of gelatin and GA, respectively^34^. A significant change in the degree of crystallinity combined with an increase in the amorphous regions of the GEL/GA_x_ copolymers was obtained^36^. This can be discussed as GA molecules decrease the ordering character in the crystalline phase of gelatin, assigning that to the crosslinking formation between the functional groups of GEL and GA molecules. In other words, complex formation within the GEL/GA_x_ composites refers to the hydroxyl groups (OH) of GA and the amino groups (NH_2_) of GEL^1^. It has been found that the crosslinking formation within the polymeric materials reduces the degree of crystallinity for some polymers^37^. Similar results were observed for gelatin and doped samples due to the interactions between gelatin and TiO_2_ nanoparticles^38,39^. There is no new peak was obtained for the GEL/GA_x_ composites. This indicates that gelatin molecules were sufficiently compatible with GA. Such results further prove the absence of a new phase within the polymeric sample. So, GA molecules can replace gelatin inter- and intra-molecular hydrogen bonds and form stable hydrogen bonds within GEL structure.

Meanwhile, FTIR spectra were used to characterize the structure of GEL/GA_x_ (x = 0, 5, 10, 15, 20, 25, and 50 wt%). Figure 3-B illustrates the FTIR spectra of pure gelatin that contains 5, 10, 15, 20, 25, and 50 wt% of GA, respectively. FTIR spectrum of gelatin exhibits several specified bands. The broad band at 3440 cm^− 1^ is attributed to the stretching vibrational of OH and the N-H stretching of amide A groups that are coupled with the strong intermolecular hydrogen bond^1^. The bands at 2920 cm^− 1^ and 2854 cm^− 1^ can be assigned to the NH_3_^+^ and C–H stretching vibrations of amide-B^2^. The bands at 1660 cm^− 1^ and 1548 cm^− 1^ could be attributed to the C = O stretching vibration of amide I and the N–H bending vibrations and C–N stretching vibrations of amide II, respectively^3^. The addition of GA to the gelatin structure leads to a shift in the band which is located at 3444 cm^− 1^ to the lower wavenumber. It was found that the OH band shifts to lower frequencies due to the formation of hydrogen bonds between the functional groups of the polymeric structure^4^. The bands at 2920 cm^− 1^ and 2854 cm^− 1^ of all GEL/GA_x_ composites shift to 2384 cm^− 1^ and 2088 cm^− 1^, respectively. This can be attributed to the covering of the functional groups of GEL and the creation of hydrogen bonds between gelatin and GA molecules^5–7^. However, the results of zeta potential measurements are given in Fig. 3-C. The zeta potential value of pure GEL recorded + 25 mV, which is agreed that obtained by^40,41^. Notably, the zeta potential value of the examined composites decreases with increasing the concentration of GA in the films till reaching negative values at concentrations of GEL/25% GA & GEL/50% GA. The zeta potential’s magnitude indicates the colloidal system’s potential stability, which means that pure gel displays more stability than GEL/ 20%GA; GEL/50%GA has more stability than GEL/25%GA. The decrease in zeta potential values implies that particles will aggregate due to the repulsion-repulsion effect, which subsequently influences the stability of the film. This result is in agreement with the previous study^40^.

### Water affinities

**Fig. 4:**
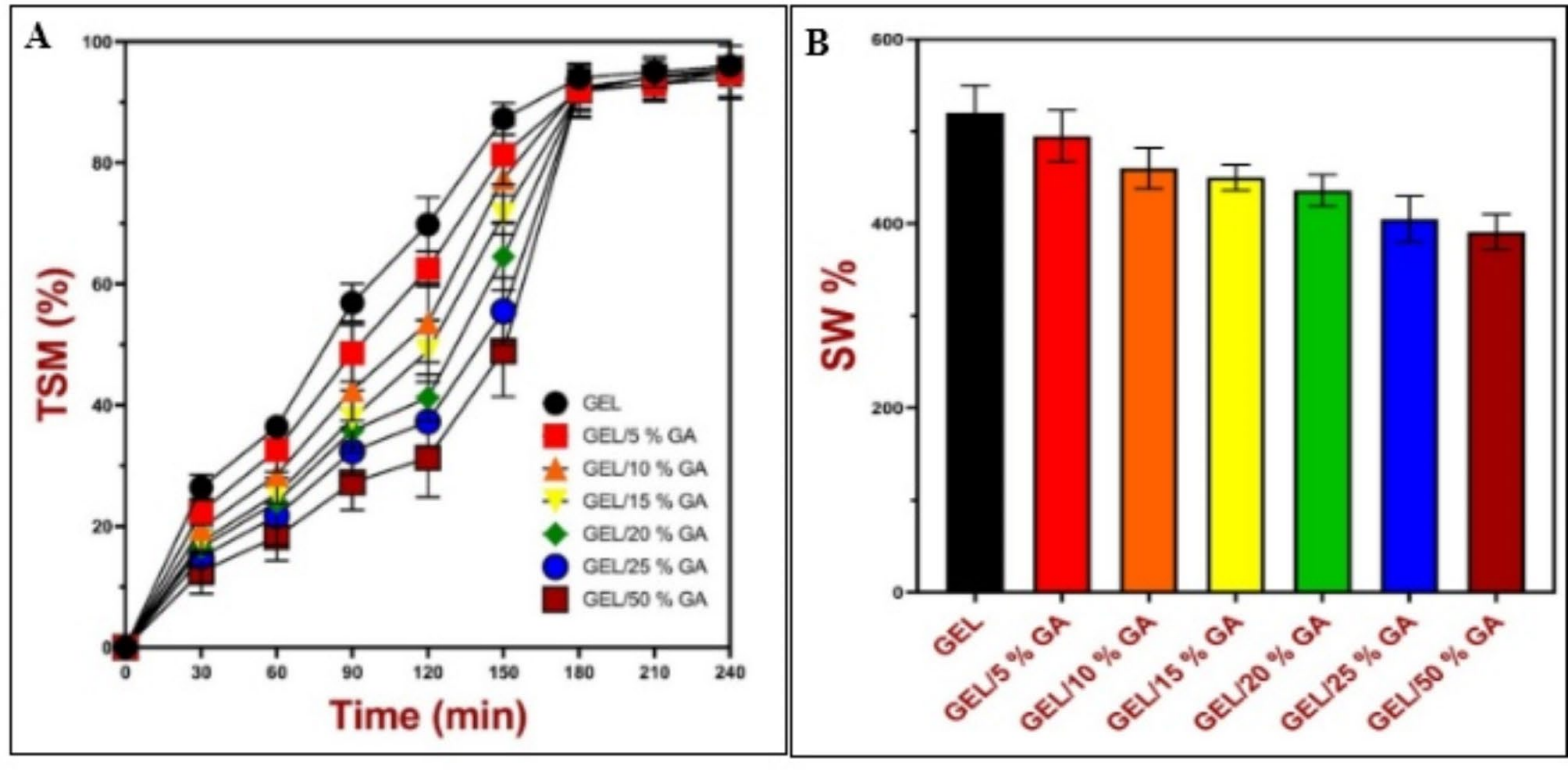
(**A**) - Kinetics of total soluble matter of the prepared GEL/GA_x_ films and (**B**) -The swelling of the prepared GEL/GA_x_ films.

The total soluble matter for all samples is obtained in Fig. 4-A which shows that the solubility increased over time till reached 100% at 240 min. The results also show that the solubility rate of GEL displayed higher hydrophobicity than samples containing GA. Wherein, the uplifting in GA percentage increases the hydrophilic level. As shown by^42,43^, the analysis of water affinities demonstrated that the WSSP75/GEL25 film (less swellable and soluble) was less hydrophilic than the GAR-based carrier. The solubility of the melanin-added films decreased as the concentration of melanin increased from 0.1 to 1% (*p* < 0.05). In general, high-water solubility may indicate lower water resistance, and the lower water solubility of melanin-modified films might result from the stronger structure of the film network via strong interactions between the protein and hydroxyl groups of melanin. The incorporation of melanin might be associated with its hydrophobic moieties. Non-polar moieties of melanin interacted favorably with the hydrophobic domains of gelatin, leading to an increase in the hydrophobicity of the resulting film.

The degree of swelling obtained from the different types of films is shown in Fig. 4-B. The films were withdrawn from the water, then the swelling, which showed the highest swellability for GEL with decreasing in its percentage through the incorporation of GA. This result is consistent with the swelling ratios of type-B gelatin films, which were remarkably higher than those of type-E gelatin films. The swelling ratios of gelatin (B1, B2, B3, E1, and E2) films increased rapidly over time within 100 min, then gelatin films gradually increased until the swelling equilibrium dissolved and disappeared^44^.

### Antimicrobial activity of as-prepared films formulated of GEL and its combination with GA

Microorganisms, which occupy Earth’s biosphere, have played a double-edged pivotal role, not only in conserving the ecosystem’s balance but also in imperiling human beings and other living creatures by their pathogenicity. Disease-causing microorganisms disseminate widely across the globe via several routes including, hospitals (e.g., medical devices), industry (e.g., food manufacturing machines), cattle farms (e.g., the effluent of fecal matter and slaughtering houses), and wastewater/drinking water treatment systems. As a consequence, innumerable antagonistic agents had evolved to restrict the spread of disease-causing microorganisms. However, the excessive, indiscriminate, and frequently inappropriate utilization of such biocides led to the prevalence of multi-drug-resistant microbes (MDR), which considers being an environmental stalemate. Remarkably, the recent and/or even traditional antimicrobial agents exhibited several limitations like toxicity to human beings and ambient milieu and insufficient efficiency against MDR. Consequently, the recent progress in the utilization of natural polymers has gained momentum because of their unique functionality, safety, and biological compatibility in an un repugnant manner to the environment. Therefore, in a trial to find a naturally efficient biocidal agent, the current study was undertaken.

**Fig. 5:**
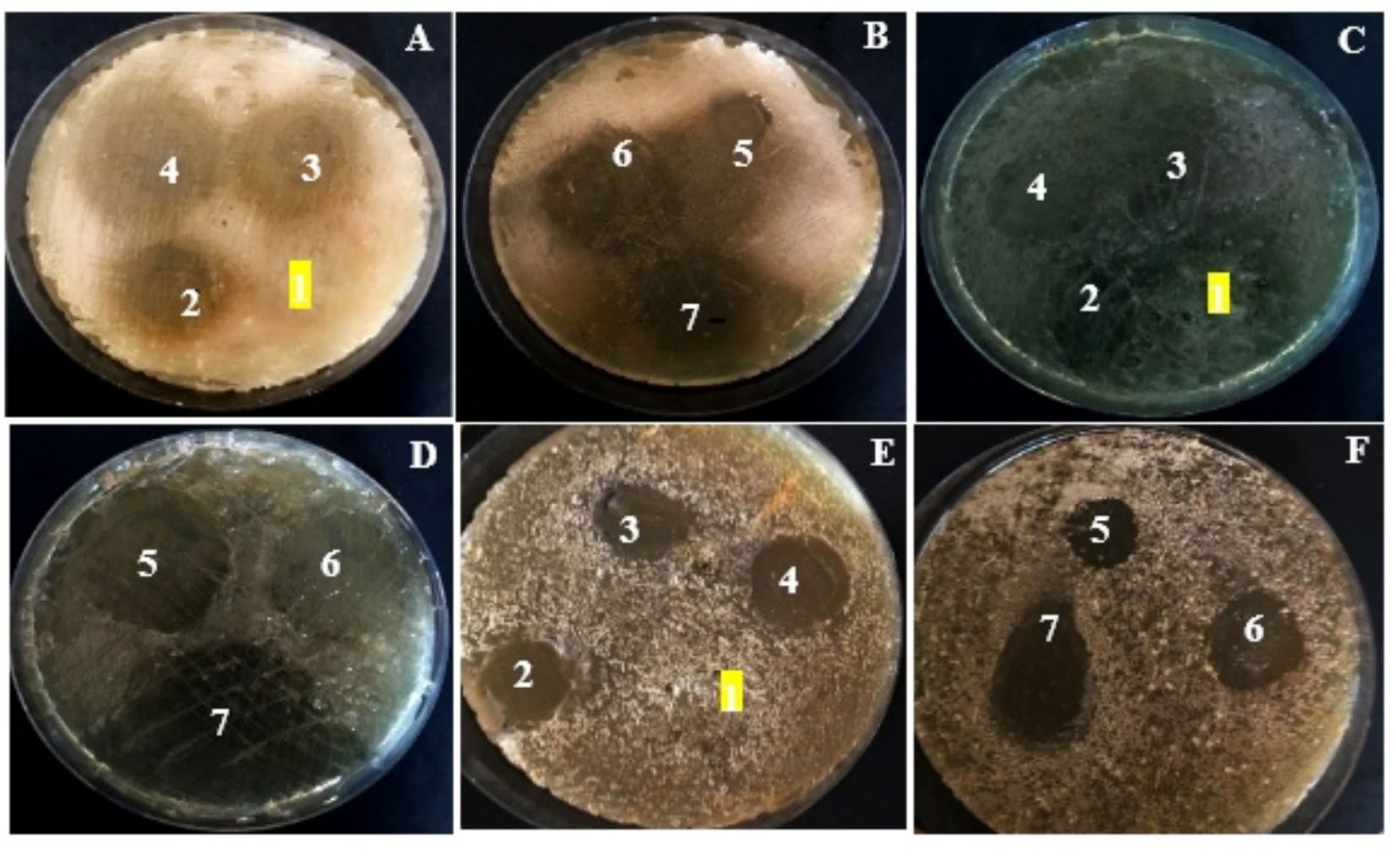
Antimicrobial activity of GEL in combination with different concentrations of GA versus different pathogens. (**A** and** B**)- *B. cereus* as a representative of Gram-positive bacteria, (**C** and** D**)-K. *pneumoniae* as a representative of Gram-negative bacteria and (**E** and** F**) *C. albicans* as a representative yeast model of examined eukaryotes. 1-symbolizes GEL, 2- symbolizes GEL/5% GA, 3- symbolizes GEL/10% GA, 4- symbolizes GEL/15% GA, 5- symbolizes GEL/20% GA, 6- symbolizes GEL/25% GA and 7- symbolizes GEL/50% GA.

Herein, the antimicrobial activity of GEL as a function of GA’s different content was investigated using disc diffusion assay versus some human pathogens, as illustrated in Table 1; Fig. 5. The selected pathogens symbolize reference strains for gram-negative, gram-positive bacteria, and unicellular and multicellular fungi; they represent the most common opportunistic pathogens causing elevated morbidity and mortality rates in the world^45^. They indwell aquatic environments and colonize several biotic and abiotic surfaces. They are the main reason for water-borne infections, food intoxication, and community-acquired and nosocomial infections^46–49^. Their pathogenicity ranged from minor skin infections (e.g., lesions and ear ulcers), passing through moderate allergy, and ending with a life-threatening illness such as severe asthma, bacteremia, endocarditis, osteomyelitis and postsurgical wound infections.

As noticed, the capability of GEL and its GA combination formulas to cease microbial proliferation varied significantly. Some of the examined pathogens seem vividly tolerant and were not influenced by all tested polymers e.g.,* (A) bracelleuse*; indicated by the absence of a clear zone. Meanwhile, obvious inhibition was recorded by *(B) cereus* in the range of 5.65 ± 0.44 to 17.2 ± 0.69 mm. A moderate inhibition effect was noticed against *P. aeruginosa* and *(C) albicans*. Therefore, the potential of GEL and its GA combination formulas for antagonizing some pathogens could be described as microbe dependent. Accordingly, it could be concluded that the sensitivity order of the tested pathogens toward the different formulations is *B. cereus > S. aureus > K. pneumoniae > P. aeruginosa > C. albicans > A. bracelleuse*. Remarkably, such variation in microbial sensitivity patterns could be attributed to the differences in the penetration rate of GA-active ingredients owing to differences in cell wall architecture and compositional organization among the examined pathogens. Where, the gram-positive bacteria differ from gram-negative bacteria in their cell wall composition and polarity. Remarkably, despite gram-positive bacteria possessing more layer number and thicker peptidoglycan (30–100 nm) compared to that gram-negative bacteria (< 10 nm), they showed higher penetrability, so, allow the diffusion of several molecules due to their vulnerable coarse meshwork^50–52^.

**Table 1:** The antagonistic activity of GEL with different GA concentration formulas via a maximum zone of inhibition (mm) against some prokaryotic and eukaryotic pathogens.

Examined pathogen	GEL	GEL/5%GA	GEL/10%GA	GEL/15%GA	GEL/20%GA	GEL/25%GA	GEL/50%GA
*B. cereus (ATCC 7464)*	0	5.65 ± 0.44	9.5 ± 0.59	10.25 ± 0.35*	11.8 ± 0.39*	13.25 ± 1.05	17.2 ± 0.69*
*S. aureus (ATCC 25923)*	0	3.9 ± 0.39	5.6 ± 0.19*	6.2 ± 0.29	6.85 ± 0.25*	7.4 ± 0.6	8.3 ± 0.29*
*P. aeruginosa (ATCC 27853)*	0	0.85 ± 0.049	1.2 ± 0.09	2.2 ± 0.09	3.45 ± 0.15	4.15 ± 0.1	5.2 ± 0.1
*K. pneumoniae (ATTC 700603)*	0	0.25 ± 0.49	0.6 ± 0.09	1 ± 0.1	1.35 ± 0.15	2.4 ± 0.3*	4.15 ± 0.64*
*C. albicans (ATCC 10231)*	0	0	0.6 ± 0.0	1 ± 0.0	1.35 ± 0.05	2.75 ± 0.25	4.1 ± 0.19
*A. bracelleuse (ATCC 16404)*	0	0	0	0	0	0	0

All values were demonstrated as mean ± SEM with significance at *p < 0.05.

Besides, gram-negative bacteria are characterized by the presence of an additional outer lipopolysaccharide membrane, which enables them to resist antimicrobial agents via modifying its hydrophobic properties and attaining mutations in porins; while gram-positive bacteria are deprived of this vital layer^53^. More so, this lipopolysaccharide layer is recognized by its negatively charged nature, which assisted in the repealing of negatively charged GEL/GA_x_ particles, especially in lower concentrations. Thus, hampering the binding of polymer molecules on gram-negative cell surfaces. Consequently, to eradicate the growth of gram-negative bacteria, higher concentrations of the GEL/GA_x_ formula are entailed. Regarding the fungal cell wall, it consists of glucans, chitin, and chitosan conjugated with glycosylated protein; however, there is heterogeneity in its structural organization among different species in the fungal kingdom^54^. Interestingly, the chitin core represents 10–20% of dry weight in filamentous or multicellular fungi and 1–2% in unicellular fungi or yeast, which could explain the higher tolerance of *A. bracelleuse* than *C. albicans*. Besides, the main core of *Candida sp.* outer layer cell wall is composed of highly branched mannans linked with mannoproteins and are not covalently bound to the glucan-chitin core, which is less rigid, thus, may exert an influence on the resistance performance of yeast cells^54^. It is worth mentioning that the differences in microbial physiology, metabolism, and cell structure could be also considered other intrinsic factors that managed the resistance/sensitivity variations among inter and intra-species of the microbes in their response to the examined composites formulas. Accordingly, the results of the present investigation will be taken into consideration in developing novel antimicrobial formulations to control candidiasis and other pneumatic infections among immunosuppressed patients after their post-infection with COVID-19 infection.

Further, the susceptibility of pathogens to the polymers increased gradually with elevating the concentration of GA associated with GEL, reflecting biocidal potency in the context of a GA dose-dependent manner. The suppression of microbial growth increased significantly by 3, 2.1, 16.6, 6.1 and 6.8-fold for *B. cereus*,* S. aureus*,* K. pneumoniae*,* P. aeruginosa*, and *C. albicans*, respectively at the highest concentration of GA (50%); when compared to the lowest concentration of GA (5%). Similarly, Al-Behadliy et al., found that 50 mg/0.1 ml of GA inhibited *E. coli* and *S. aureus* growth by 12.9 and 12.6 mm ZOI; whereas, it assessed by 6.36 and 7.7 mm at 10 mg/0.1 mL^30^.

### Disinfection of real wastewater samples

Microbial contamination of the aquatic environment threatens the quality of water bodies and their sanitary state, which subsequently adversely on drinking water supply, irrigation, industrial, urbanization, and recreational activities, especially with the continuous increase in population. Remarkably, it originated from effluents that were discharged from water treatment plants, decontamination stations, domesticated animals (manure spreading, pit stock overflow), hospitals, and industries^55^. Upon consuming microbially-polluted drinking water, particularly in developing countries, major public health concerns such as endemic gastroenteritis are generated^56^. Subsequently, due to the water crisis problem, the utilization of adequate disinfection practices to decontaminate polluted water to standards limits is an urgent requirement. That also would enable the safe recruitment of treated water that was recovered from wastewater treatment plants. While chlorination is the most popular and effective traditional approach that was used for disinfection purposes, its multiple drawbacks entail endeavoring other safe green alternatives like natural polymers. Therefore, the disinfection potency of GEL and its GA combination formulas (3 and 10% W/V) was scrutinized in two real wastewater samples. As noticed in Tables 2 and 3, the pristine GEL didn’t offer any disinfection potential. Conversely, the association of different concentrations of GA increased progressively the microbial inhibitory power in both types of effluents.

**Fig. 6:**
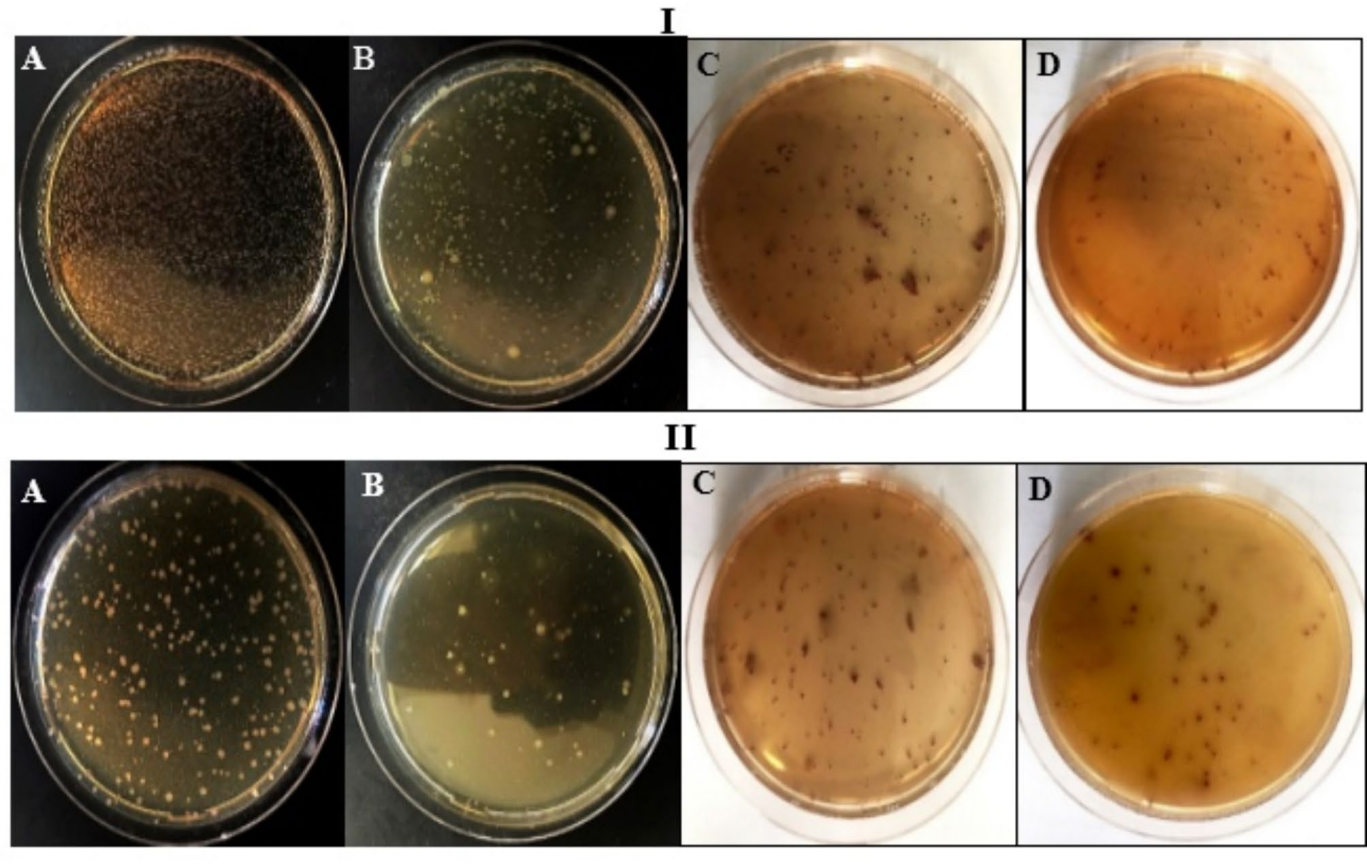
Antagonistic activity of GEL supplemented with 50% GA against total bacterial content and coliform-dwelling municipal effluent (I) and industrial effluent (II). (**A**)- TPC of the untreated sample, (**B**)- TPC after treatment with 10% of the polymer, (**C**)- coliform before treatment, and (**D**)-Coliform after treatment with 10% of the polymer.

Empirically, the microbial content of untreated or control municipal effluents was assessed by 74.45 × 10^9^ ± 3.2 × 10^8^; upon employing different concentrations of GA in conjugation to GEL films, the microbial load decreased gradually till reached the maximum inhibition recording 46.91 × 10^9^ ± 8.1 × 10^7^ and 40.48 × 10^9^ ± 1.1 × 10^8^ CFU/mL for 3 and 10% of GEL integrated with 50% GA content in municipal effluents, which equivalate 36.9 ± 1.0.9 and 45.62 ± 1.48% disinfection potency. Regarding the industrial effluent, lower inhibitory power was exhibited by all examined polymer formulations in their response to the microbial load, relative to that observed in the municipal sample. That could be ascribed to the resistance capability of indigenous microbiota that was developed due to the enrichment of industrial effluents with various heavy metal pollutants, as accentuated by physicochemical studies^31^. Meanwhile, the higher organic load and suspended solid content in effluents might hinder the better attachment of antimicrobial agents with microbial surfaces and eventually lower suppression potential. The TPC of industrial effluents estimated at 4.075 × 10^4^ ± 7.4 × 10^2^ and 3.439 × 10^4^ ± 9.0 × 10^2^ CFU/mL after treatment with both concentrations of GEL integrated with 50% GA content, compared to the untreated sample which recorded 5.501 × 10^4^ ± 2.2 × 10^2^ CFU/mL. Virtually, the disinfection potency of GEL integrated with 50% GA content reached 25.9 ± 1.35 and 37.48 ± 1.63% for both examined doses, respectively (Fig. 6).

**Table 2:** Disinfection potency of GEL as a function of GA concentrations (3 and 10%) on TPC, coliform count, and metabolic activity of municipal effluent. All values were demonstrated as mean ± SEM with significance at *p < 0.05.

Examined Polymers	Dose	Municipal		
		TPC (CFU/mL)	Coliform count (CFU/mL)	Metabolic activity (O.D)
Control	–	74.45 × 10^9^ ± 3.2 × 10^8^	12.13 × 10^4^ ± 1.7 × 10^2^	1.0 ± 0.01
GEL	3%	75.51 × 10^9^ ± 1.8 × 10^8^	12.134 × 10^4^ ± 45	1.01 ± 0.01
	10%	75.95 × 10^9^ ± 8.5 × 10^7^	12.179 × 10^4^ ± 54	1.019 ± 0.02
GEL/5%GA	3%	71.84 × 10^9^ ± 4.5 × 10^8^	12.05 × 10^4^ ± 86	0.942 ± 0.03
	10%	70.26 × 10^9^ ± 9.2 × 10^8^	11.65 × 10^4^ ± 1.1 × 10^2^	0.911 ± 0.07
GEL/10%GA	3%	68.59 × 10^9^ ± 1.1 × 10^8^	11.63 × 10^4^ ± 73	0.881 ± 0.05
	10%	64.89 × 10^9^ ± 1.2 × 10^8^	10.93 × 10^4^ ± 1.9 × 10^2^	0.832 ± 0.15
GEL/15%GA	3%	63.09 × 10^9^ ± 1.3 × 10^8^	11.13 × 10^4^ ± 1.1 × 10^2^	0.810 ± 0.15
	10%	59.36 × 10^9^ ± 1.3 × 10^8^	9.778 × 10^4^ ± 2.1 × 10^2^	0.759 ± 0.13
GEL/20%GA	3%	58.11 × 10^9^ ± 2.1 × 10^8^	10.35 × 10^4^ ± 91	0.738 ± 0.20
	10%	53.11 × 10^9^ ± 1.6 × 10^8^	8.411 × 10^4^ ± 1.1 × 10^2^	0.682 ± 0.14*
GEL/25%GA	3%	54.61 × 10^9^ ± 3.5 × 10^8^*	9.415 × 10^4^ ± 1.4 × 10^2^	0.704 ± 0.14
	10%	48.74 × 10^9^ ± 1.6 × 10^8^	7.147 × 10^4^ ± 2.3 × 10^2^*	0.590 ± 0.11*
GEL/50%GA	3%	46.91 × 10^9^ ± 8.1 × 10^7^*	6.814 × 10^4^ ± 1.6 × 10^2^*	0.572 ± 0.09
	10%	40.48 × 10^9^ ± 1.1 × 10^8^*	5.041 × 10^4^ ± 2.5 × 10^2^*	0.420 ± 0.22*

In addition, the coliform group is typically employed as an indicator of water microbial pollution caused mainly by the fecal material of human beings and warm-blooded animals. Reckon on standards, environmental guidelines, and maximum allowable concentrations, which define the water quality variables, the suitability of water for safe human uses is determined by detecting and enumerating the coliforms group^57^. Hence, the assessment of both effluents’ quality after disinfection with different doses of polymer formulations has proceeded. As noticed previously in total plate count, the coliform count decreased in a dose-dependent way. Besides, the effectiveness of all examined polymers seemed to be more efficient in ceasing the coliform growth in municipal effluent than that harboring industrial effluent. The highest significant inhibition of the coliform growth (58.43 ± 2.07 and 40.88 ± 2.24%) was achieved by GEL integrated with 50% GA at the highest dose, which recorded 5.041 × 10^4^ ± 2.5 × 10^2^ and 7.02 × 10^2^ ± 26.62 CFU/mL relative to the untreated samples of 12.13 × 10^4^ ± 1.7 × 10^2^ and 11.885 × 10^2^ ± 5.5 CFU/mL for municipal and industrial wastewater, respectively. Ultimately, this outcome infers that the applied doses of GEL/GA_x_ formulations were not enough to exhibit complete elimination of microbial loads in both examined wastewater samples, which entails further optimization stage for the applied dose and contact time. Therefore, it is worth mentioning that each kind of wastewater requires its designed dose of antimicrobial agent to exert a tangible effect in the disinfection process. The appropriate adjustment of this dose relies on the quality criteria of examined wastewater, which encompasses pH, turbidity, temperature, total suspended solids, ionic strength, organic matter content, heavy metal content, microbial load, biological oxygen demand, and chemical oxygen demand^58^.

**Table 3:** Disinfection potency of GEL as a function of GA concentrations (3 and 10%) on TPC, coliform count, and metabolic activity of industrial effluent. All values were demonstrated as mean ± SEM with significance at *p < 0.05.

Examined Polymers	Dose	Industrial effluent		
		TPC (CFU/mL)	Coliform count (CFU/mL)	Metabolic activity (O.D)
Control	–	5.501 × 10^4^ ± 2.2 × 10^2^	11.885 × 10^2^ ± 5.5	1.000 ± 0.003
GEL	3%	5.533 × 10^4^ ± 0.52 × 10^2^	11.8867 × 10^2^ ± 0.047	1.0002 ± 0.003
	10%	5.572 × 10^4^ ± 0.11 × 10^2^	11.9195 × 10^2^ ± 1.069	1.0038 ± 0.03
GEL/5%GA	3%	5.399 × 10^4^ ± 3.9 × 10^2^	11.8381 × 10^2^ ± 2.91	0.990 ± 0.151
	10%	5.322 × 10^4^ ± 2.8 × 10^2^	11.6425 × 10^2^ ± 3.44	0.965 ± 0.349
GEL/10%GA	3%	5.286 × 10^4^ ± 5.0 × 10^2^	11.5456 × 10^2^ ± 6	0.960 ± 0.149*
	10%	5.088 × 10^4^ ± 6.2 × 10^2^	11.0203 × 10^2^ ± 12.89	0.901 ± 0.179
GEL/15%GA	3%	5.159 × 10^4^ ± 5.1 × 10^2^	11.2147 × 10^2^ ± 6.53	0.916 ± 0.178
	10%	4.963 × 10^4^ ± 8.7 × 10^2^	10.6644 × 10^2^ ± 10.81	0.855 ± 0.119
GEL/20%GA	3%	4.928 × 10^4^ ± 7.0 × 10^2^	10.7434 × 10^2^ ± 15.15	0.838 ± 0.114
	10%	4.557 × 10^4^ ± 5.6 × 10^2^	9.4978 × 10^2^ ± 19.9	0.759 ± 0.138
GEL/25%GA	3%	4.701 × 10^4^ ± 4.5 × 10^2^	10.1759 × 10^2^ ± 25.79	0.765 ± 0.136
	10%	4.189 × 10^4^ ± 1.2 × 10^2^	8.6808 × 10^2^ ± 16.75	0.695 ± 0.104*
GEL/50%GA	3%	4.075 × 10^4^ ± 7.4 × 10^2^	8.4847 × 10^2^ ± 23.41	0.628 ± 0.171*
	10%	3.439 × 10^4^ ± 9.0 × 10^2^*	7.02 × 10^2^ ± 26.62*	0.496 ± 0.174*

Moreover, the antagonistic potentiality of examined formulas extended to prohibit the microbial metabolic activity, in both examined types of wastewater, as revealed by the MTT assay (Tables 2 and 3). Similarly, the formula of GEL supplemented with 50% GA, at a concentration of 10% W/V, succeeded in minimizing the microbial metabolism significantly by 57.91 ± 2.27 and 50.37 ± 1.74% in municipal and industrial wastewater, respectively; implying the effectiveness of examined formulas in blocking the viability and proliferation of other microbial forms including unculturable microbes. The proposed explanation could be deduced from the differences in the values of suppression potential percentage in both TPC and metabolic activity by MTT. Where the inhibition percentage of microbial activity exceeds that resulted from TPC.

According to the former results, this study displays the importance of incorporating various doses of GA in GEL films and recruiting such composite formulas as antimicrobial agents and disinfectants. Whereas gelatin as a proteinaceous material didn’t exert any biocidal activity. Therefore, it was employed for coating and adhesion purposes. It is characterized by its bio-based/ biodegradable nature, biocompatibility, non-toxicity, adequate gelling traits, dissolution behavior, high cross-linking activity, and stable emulsions. Its structure encompasses a high content of proline, hydroxyproline, and glycine which assist in the production of flexible films^59^. Nonetheless, its deficient antimicrobial potency, low mechanical strength, and high-temperature sensitivity may restrict its applications^60^. Thus, other natural biopolymers such as GA, chitosan, starch, cellulose, nanoparticles, essential oils, and antimicrobial agents were added to it to improve the overall physical and biological properties^59–64^. Herein, the amalgamation of GEL with GA generated potent antagonistic composites, where the merits of each component compensate for the shortcomings of another one.

So, it is plausible to state the outstanding properties of GA. As a water-soluble gummy Acacian exudate, it is a branched amphiphilic hetero polysaccharide characterized by its colorless feature, low viscosity, good retention of volatiles, high solubility, negatively charged properties, and antioxidant activity. Its structure of hydrophilic polysaccharides mixture (44% of galactopyranose, 25% of arabinopyranose, arabinofuranose, 14% of rhamnopyranose, 15.5% of glucuropyranosyl uronic acid and1.5% of methyl glucuropyranosyl uronic acid) and glycoproteins (1–3%) facilitated its usage in microencapsulation and emulsification^64^. However, its content ratios and physicochemical properties vary according to the age of the trees, geography, soil condition, and climatic condition^65^. Besides, it contains phenolic compounds and other metals such as sodium, calcium, potassium (41.98 to 47.23 µg/g), and a high level of iron (24.02 to 31.79 µg/g)^66,67^.

Seemingly, the tightly bounding of flexible positively charged amino acids of GEL with negatively charged polysaccharide of GA, as indicated by zeta-potential (Fig. 3-C), provided a considerable polydispersity and better contact with microbial functional groups, which eventually enhanced the biocidal capability. Arguably, GEL supplied GA with an adequate matrix that enhanced its penetration strength. Thus, it contributed indirectly to the biocide powerful of GA. Strikingly, the presence of hydrophobic branches in GA probably participated in the eminency of its antimicrobial activity and disinfection potency. As the hydrophobic polypeptide chains of GA adsorb and anchored onto the lipopolysaccharide layer of the microbial outer cell wall as a barrier; thereby, influencing cell permeability, allowing the passage of other GA hydrophilic components into the cell interiorly and causing pits in the cell wall, destabilize cell membrane and ultimately leakage of intracellular essential elements. As revealed by Kopiasz et al., and Tyagi & Mishra, polymer average molecular weight, type and distribution of cationic/anionic groups, molecular architecture (homopolymer, branched polymers, random or block copolymer, etc.), amphiphilic balance (hydrophilicity/lipophilicity) and polymer aggregation could manage the biological activity of any polymer-polymer interaction^68,69^.

**Fig. 7:**
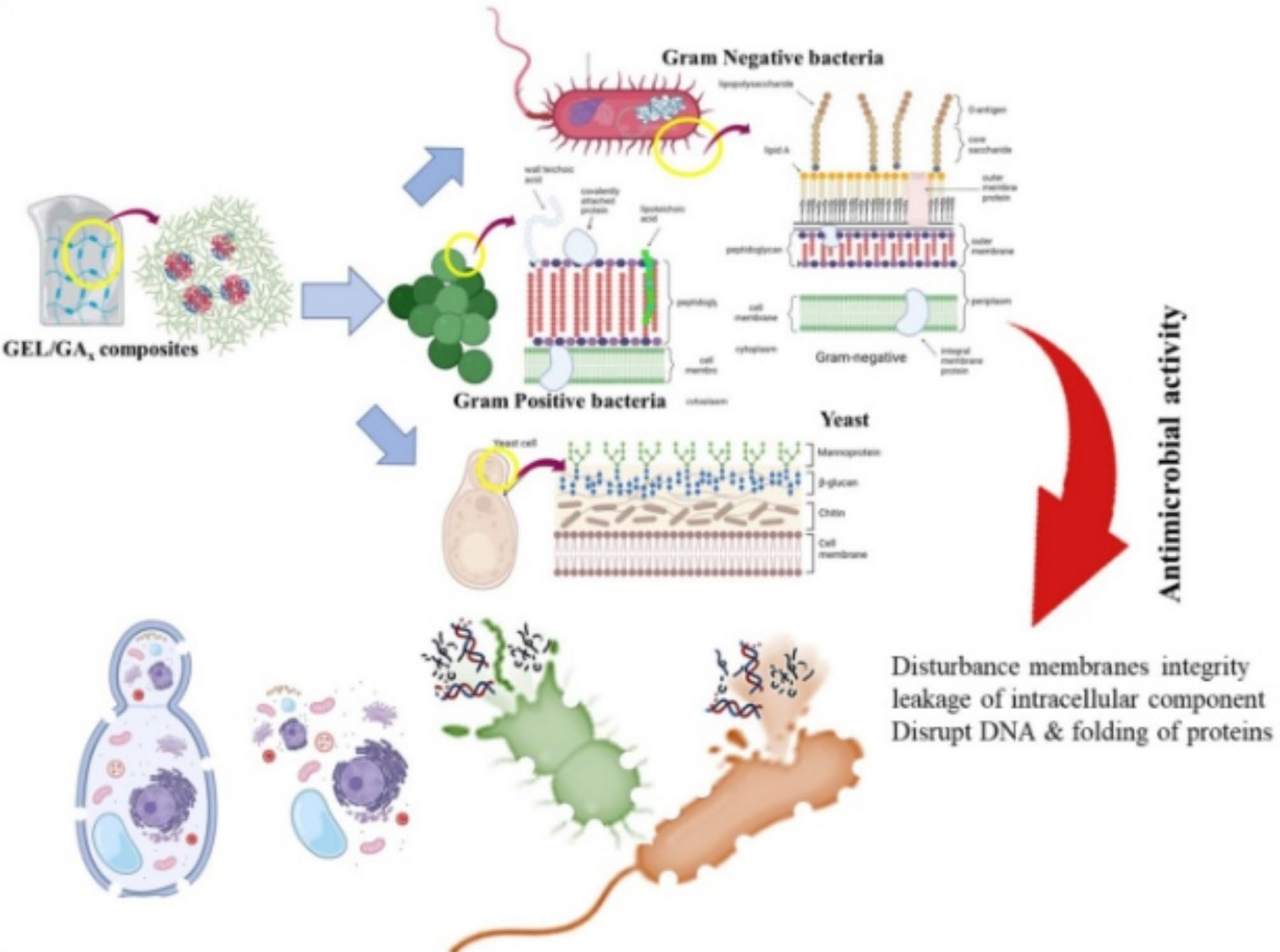
Graphical representation of antimicrobial mechanism exerted by GEL/GA_x_ against different pathogens.

In agreement with our results, Mohamed et al. found that 0.5 and 1% of GA inhibited the growth of gram-positive bacteria more than gram-negative attributing that to the disturbance caused by GA on cell membranes integrity, imbalance in the osmotic pressure of inner and outer sides of the cell wall, which finally led to the external leakage of water and another soluble intracellular component^70^. Besides, GA also involved some heavy metals such as arsenic, lead (˂ 20 ppm), phenolic compounds (e.g., flavonoids, alkaloids, saponin, and sterols/triterpenoids), and enzymes such as pectinase, peroxidase and oxidase, which could also exert a remarkable biocidal activity by disrupting the functionality of vital biomolecules^71^. The antimicrobial property of GA against several bacterial and fungal species were detected in several works of literature^72,73^. Additionally, the acidic nature of GA (pH = 4.45–4.9) also may play a vital role in this antagonistic potentiality^42^. In this way, the acidic soluble GA components that penetrate inside the cells would acidify the cytoplasm, collapse proton gradients, alter functional groups of microbial biomolecules, disrupt the folding of proteins, inactivate enzymes, and influence adversely on gene regulation^74^. Figure 7 demonstrates the graphical illustration of the proposed antimicrobial mechanism followed by GEL/GA_x_ to suppress the microbial growth. Generally, the unspecific inhibitory mechanism followed by GEL-GA-based antimicrobial films of the present study, may target multiple sites simultaneously at the cellular level; implying the possibility to inhibit microbial growth with less susceptibility to resistance development, compared to traditional antibiotics.

Notably, gelatin as a protenaceous matrix was utilized in various gelatin-based composite fibrous membranes to be employed in medical application. That was implemented through the hybridization with chitosan, graphene oxide and hydroxyapatite to fabricate multi-functional biomimetic scaffolds suitable for bone scaffold applications^75^. Similarly, Kreller et al.^76^ modified the structure of GEL to form Alginate – GEL hydrogel for cartilage tissue engineering purpose. Meanwhile, GEL-based hydrogels with microcrystalline and nanocrystalline cellulose were recruited in food packaging application^77^. While (Sarwar et al.) and (Perumal et al.) fabricated GEL-chitosan membranes for desalination and heavy metal removal^78,79^. Other scholars categorized GEL-bases composites/nanohybrides for remediating wastewater from various contaminants^80–82^. On the other hand, GA-based composites were prepared and incorporated with cellulose, attapulgite (Wang et al.), polyvinylchloride (Aji et al.), polyacrylamide / polyacrylic acid (Elbedwehy et al.) and nanomaterials such as TiO_2_ (Lopes et al.), Fe_3_O_4_ (Vatanpour et al.) to remediate water from organic dyes, heavy metals and biofouling^83–87^. Likewise, recent literatures review listed the diverse GA-based polymers/nanocomposites for wide array of medical and environmental applications^88,89^.

Intriguingly the disinfection potency of our designed GEL/GA_x_ composites were not scrutinized before, according to our acquaintance, which triggered our study advantageous. From our perspective, the recruitment of our protein-carbohydrate assembly in wastewater disinfection, at water purification plants, and for effluents from different industries, before discharge into water bodies is considered a promising alternative solution to traditional treatment means. Let al.one the possibility of being applied in the food industry (packaging, additive, coating, antioxidant, coloring agents, emulsifiers, etc.). Wherein, both components had been approved by the US Food and Drug Administration. Medically and pharmaceutically, GA was extensively employed in the treatment of, burns, wounds, inflammation of intestinal mucosa, coughing, sore throat, diarrhea, dysentery, and urinary tract ailments^73^. Consequently, in this context, the employing of GEL-based GA films would find an avenue in tissue engineering (e.g., bone, ligament, cartilage, heart, nerves, etc.), wound dressings/adhesives and controlled drug delivery by the virtue of their biological traits including cytocompatibility, osteoconductive properties, non-immunogenicity, non-antigenicity and also physicochemical traits such as plasticity, adhesiveness, dissolution and flexibility in processing (e.g., films, scaffolds, fibers, gel, ointment, cream, dressing).

Recently, the combination or adjuvant therapy represents an effective and promising therapeutic solution or even prophylactic strategy to frustrate MDR infections via utilizing two antimicrobial ingredients in a synergistic manner^90^. The incorporation of other biocidal materials such as drugs, nanoparticles, antioxidants, plant extracts, and antimicrobial peptides in protein/polysaccharide-based antimicrobial polymers has been reported^91–93^. Finally, the current study succeeded in the establishment of a basic stone for the future applicability of the optimized green protein-carbohydrate system with further modifications (e.g., novel nanoparticles, antimicrobial peptides, probiotics, and anticancer drugs) to be invested in water treatment machines, anti-biofouling membranes, food packaging, dietary supplements, wound /Acne -healing and cancer therapy.

## Conclusion

In the current study, different composites of GEL-based GA were prepared in different concentrations of GA (i.e., 5, 10, 15, 20, 25, and 50%); subsequently characterized by different techniques: SEM, XRD, FTIR and *ζ-*potential. The results reflected the enhancement in the physiochemical properties of the designed formulas upon elevating GA concentration to 50%. Wherein, the miscibility, homogenous structure, stability, solubility and swellability degrees were improved as revealed by the water affinity data. Additionally, the antagonistic potentiality of the designed formulas against prokaryotic and eukaryotic pathogens were scrutinized. The results followed microbe-dependent manner in sensitivity order of *B. cereus > S. aureus > K. pneumoniae > P. aeruginosa > C. albicans > A. bracelleuse.* However, the maximum inhibition was noticed in the range of 4.1 ± 0.19 to 17.2 ± 0.69 mm at 50% content of GA. Meanwhile, the formula of GEL/50%GA showed the superior capability to disinfect municipal and industrial effluents with inhibition percentage of TPC, coliforms and microbial activity reached to 45.62 ± 1.48, 58.43 ± 2.07% and more than 50%, respectively, reflecting promising results in wastewater treatment systems. For the GEL/GA_x_ to be used on a large commercial scale, further studies should be done to improve the mechanical and water solubility properties of GEL/GA_x_ composites alone or with the addition of other supporting materials.

## Data Availability

Data is provided within the manuscript.
